# Chlorido(pyridine-2-carbaldehyde oximato-κ^2^ *N*,*N*′)(pyridine-2-carbaldehyde oxime-κ^2^ *N*,*N*′)copper(II)

**DOI:** 10.1107/S1600536808014748

**Published:** 2008-05-21

**Authors:** Genhua Wu, Dayu Wu

**Affiliations:** a School of Chemistry and Chemical Engineering, Anqing Teachers College, Anqing 246011, People’s Republic of China

## Abstract

In the title compound, [Cu(C_6_H_5_N_2_O)Cl(C_6_H_6_N_2_O)], the Cu atom is coordinated by one neutral and one deprotonated pyridine-2-carboxaldehyde oxime (pco) ligand, resulting in the formation of two five-membered CuN_2_C_2_ rings. Together with the additional coordinating chloride anion, the coordination polyhedron of copper is best described as a distorted square-pyramid, the distortion parameter being 0.288. The two organic ligands are linked by an intramolecular O—H⋯O hydrogen bond.

## Related literature

For related literature, see: Addison *et al.* (1984 ▶); Afrati *et al.* (2005 ▶); Korpi *et al.* (2005 ▶); Pearse *et al.* (1989 ▶); Stamatatos *et al.* (2006 ▶).
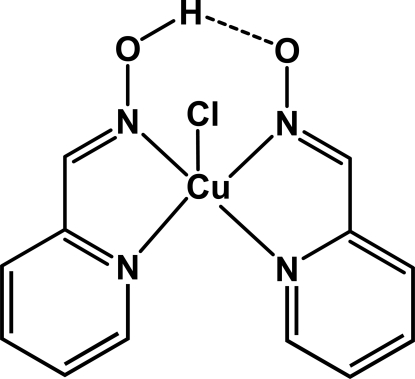


## Experimental

### 

#### Crystal data

[Cu(C_6_H_5_N_2_O)Cl(C_6_H_6_N_2_O)]
*M* *_r_* = 342.24
Monoclinic, 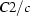

*a* = 16.686 (2) Å
*b* = 12.064 (2) Å
*c* = 13.805 (1) Å
β = 109.02 (1)°
*V* = 2627.3 (5) Å^3^
*Z* = 8
Mo *K*α radiation
μ = 1.87 mm^−1^
*T* = 293 (2) K
0.22 × 0.18 × 0.15 mm

#### Data collection

Bruker SMART CCD area-detector diffractometer
Absorption correction: multi-scan (*SHELXTL*; Sheldrick, 2008 ▶) *T*_min_ = 0.488, *T*_max_ = 0.594 (expected range = 0.620–0.755)
6487 measured reflections
2318 independent reflections
1788 reflections with *I* > 2σ(*I*)
*R*_int_ = 0.034

#### Refinement

*R*[*F* ^2^ > 2σ(*F* ^2^)] = 0.029
*wR*(*F* ^2^) = 0.110
*S* = 1.01
2318 reflections
181 parameters
H-atom parameters constrained
Δρ_max_ = 0.40 e Å^−3^
Δρ_min_ = −0.39 e Å^−3^

### 

Data collection: *SMART* (Bruker, 1997 ▶); cell refinement: *SAINT* (Bruker, 1997 ▶); data reduction: *SAINT*; program(s) used to solve structure: *SHELXS97* (Sheldrick, 2008 ▶); program(s) used to refine structure: *SHELXL97* (Sheldrick, 2008 ▶); molecular graphics: *SHELXTL* (Sheldrick, 2008 ▶); software used to prepare material for publication: *SHELXTL*.

## Supplementary Material

Crystal structure: contains datablocks I, global. DOI: 10.1107/S1600536808014748/im2064sup1.cif

Structure factors: contains datablocks I. DOI: 10.1107/S1600536808014748/im2064Isup2.hkl

Additional supplementary materials:  crystallographic information; 3D view; checkCIF report

## Figures and Tables

**Table 1:** Hydrogen-bond geometry (Å, °)

*D*—H⋯*A*	*D*—H	H⋯*A*	*D*⋯*A*	*D*—H⋯*A*
O1—H1*B*⋯O2	0.82	1.70	2.488 (5)	162

**Fig. 1.**
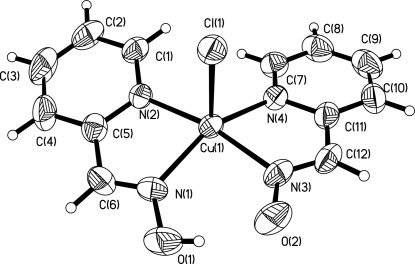
The molecular structure of the title compound with the atom-numbering scheme. Displacement ellipsoids were drawn at the 50% probability level.

## supplementary crystallographic information

### Comment

Pyridine-2-carbaldehyde oxime ligands usually bind to metals in a bidentate fashion, either chelating one metal center or bridging two metals. Their complexes find application in diverse areas such as functional supramolecular design, magnetic materials and catalysis (Korpi *et al.*, 2005; Pearse *et al.*, 1989; Afrati *et al.*, 2005; Stamatatos *et al.*, 2006). The title compound is a new copper complex from the reaction of CuCl_2_ with pyridine-2-carbaldehyde oxime (pco). The compound consists of two N,N-chelating ligands and one chloride anion. The two pco ligands are coordinated to copper to form two five-membered CuC_2_N_2_ rings. The copper atom adopts a distorted 4 + 1 square-pyramidal coordination mode with the distortion parameter being 0.288 (Addison *et al.*, 1984) and the angles around copper ion ranging from 79.07 (1)° for N3—Cu1—N4 to 168.37 (1)° for N2—Cu1—N3. From the viewpoint of charge balance, it is presumed there exists one deprotonated and one protonated oxime ligand with a strong intramolecular hydrogen bond between the OH group and the negatively charged oxygen of the other ligand (O1···O2 = 2.488 Å) which would also give an explanation for the rather unusal cis-arrangement of the ligands (Scheme 1, Figure 1. ).

### Experimental

A methanolic solution (15 ml) containing pco (0.1 mmol, 0.012 g) was added to an methanolic solution (10 ml) containing CuCl_2_ × 2 H_2_O (0.1 mmol, 0.017 g). After stirring for 2 h, the solution was filtered. Dark green needle-like crystals suitable for single-crystal X-ray diffraction were obtained by evaporating the resulting filtrate in air for several days (yield 65.6% based on the ligand).

### Refinement

H atoms were placed geometrically and allowed to ride during refinement with C—H = 0.93–0.96 Å and O—H = 0.82 Å with *U*_iso_(H) = 1.2 or 1.5Ueq(C or O).

### Crystal data

**Table:**

[Cu(C_6_H_5_N_2_O)Cl(C_6_H_6_N_2_O)]	*F*_000_ = 1384
*M**_r_* = 342.24	*D*_x_ = 1.730 Mg m^−^^3^
Monoclinic, *C*2/*c*	Mo *K*α radiation λ = 0.71073 Å
Hall symbol: -C 2yc	Cell parameters from 2343 reflections
*a* = 16.686 (2) Å	θ = 2.4–26.6º
*b* = 12.064 (2) Å	µ = 1.87 mm^−^^1^
*c* = 13.805 (1) Å	*T* = 293 (2) K
β = 109.02 (1)º	Block, dark green
*V* = 2627.3 (5) Å^3^	0.22 × 0.18 × 0.15 mm
*Z* = 8	

### Data collection

**Table:**

Bruker SMART CCD area-detector diffractometer	2318 independent reflections
Radiation source: fine-focus sealed tube	1788 reflections with *I* > 2σ(*I*)
Monochromator: graphite	*R*_int_ = 0.034
*T* = 293(2) K	θ_max_ = 25.0º
φ and ω scans	θ_min_ = 2.1º
Absorption correction: multi-scan(SHELXTL; Sheldrick, 2008)	*h* = −14→19
*T*_min_ = 0.488, *T*_max_ = 0.594	*k* = −14→13
6487 measured reflections	*l* = −16→16

### Refinement

**Table:**

Refinement on *F*^2^	Secondary atom site location: difference Fourier map
Least-squares matrix: full	Hydrogen site location: inferred from neighbouring sites
*R*[*F*^2^ > 2σ(*F*^2^)] = 0.029	H-atom parameters constrained
*wR*(*F*^2^) = 0.110	*w* = 1/[σ^2^(*F*_o_^2^) + (0.065*P*)^2^ + 1.2*P*] where *P* = (*F*_o_^2^ + 2*F*_c_^2^)/3
*S* = 1.01	(Δ/σ)_max_ = 0.001
2318 reflections	Δρ_max_ = 0.40 e Å^−^^3^
181 parameters	Δρ_min_ = −0.39 e Å^−^^3^
Primary atom site location: structure-invariant direct methods	Extinction correction: none

### Special details

**Table:**

Geometry. All e.s.d.'s (except the e.s.d. in the dihedral angle between two l.s. planes) are estimated using the full covariance matrix. The cell e.s.d.'s are taken into account individually in the estimation of e.s.d.'s in distances, angles and torsion angles; correlations between e.s.d.'s in cell parameters are only used when they are defined by crystal symmetry. An approximate (isotropic) treatment of cell e.s.d.'s is used for estimating e.s.d.'s involving l.s. planes.
Refinement. Refinement of *F*^2^ against ALL reflections. The weighted *R*-factor *wR* and goodness of fit *S* are based on *F*^2^, conventional *R*-factors *R* are based on *F*, with *F* set to zero for negative *F*^2^. The threshold expression of *F*^2^ > σ(*F*^2^) is used only for calculating *R*-factors(gt) *etc*. and is not relevant to the choice of reflections for refinement. *R*-factors based on *F*^2^ are statistically about twice as large as those based on *F*, and *R*- factors based on ALL data will be even larger.

### Fractional atomic coordinates and isotropic or equivalent isotropic displacement parameters (Å^2^)

**Table:**

	*x*	*y*	*z*	*U*_iso_*/*U*_eq_	
Cu1	0.45583 (2)	0.75857 (3)	0.08754 (3)	0.03490 (18)	
Cl1	0.39133 (6)	0.74459 (6)	−0.09737 (7)	0.0452 (3)	
N2	0.39301 (17)	0.8985 (2)	0.1014 (2)	0.0365 (6)	
N3	0.53622 (18)	0.6345 (2)	0.0978 (2)	0.0432 (7)	
N4	0.39418 (17)	0.6311 (2)	0.1352 (2)	0.0386 (7)	
O2	0.60997 (16)	0.6484 (2)	0.0801 (2)	0.0594 (7)	
C1	0.3107 (2)	0.9115 (3)	0.0892 (3)	0.0445 (9)	
H1A	0.2761	0.8491	0.0764	0.053*	
N1	0.54953 (17)	0.8713 (2)	0.1156 (2)	0.0431 (7)	
C11	0.4316 (2)	0.5328 (3)	0.1314 (3)	0.0427 (9)	
C5	0.4419 (2)	0.9892 (3)	0.1166 (3)	0.0433 (8)	
O1	0.63020 (15)	0.8491 (3)	0.1224 (2)	0.0666 (8)	
H1B	0.6346	0.7834	0.1100	0.100*	
C2	0.2750 (3)	1.0126 (4)	0.0946 (3)	0.0585 (11)	
H2A	0.2177	1.0187	0.0866	0.070*	
C9	0.3236 (3)	0.4362 (4)	0.1762 (3)	0.0647 (12)	
H9A	0.3002	0.3707	0.1903	0.078*	
C7	0.3232 (2)	0.6295 (3)	0.1599 (3)	0.0457 (9)	
H7A	0.2973	0.6968	0.1638	0.055*	
C12	0.5115 (2)	0.5390 (3)	0.1109 (3)	0.0463 (9)	
H12A	0.5426	0.4760	0.1076	0.056*	
C6	0.5299 (2)	0.9703 (3)	0.1257 (3)	0.0483 (9)	
H6A	0.5692	1.0276	0.1381	0.058*	
C10	0.3975 (3)	0.4349 (3)	0.1509 (3)	0.0579 (11)	
H10A	0.4239	0.3680	0.1471	0.069*	
C8	0.2865 (3)	0.5352 (4)	0.1798 (3)	0.0593 (11)	
H8A	0.2365	0.5388	0.1957	0.071*	
C4	0.4094 (3)	1.0927 (3)	0.1215 (3)	0.0636 (12)	
H4A	0.4440	1.1550	0.1313	0.076*	
C3	0.3253 (3)	1.1034 (3)	0.1118 (4)	0.0718 (13)	
H3A	0.3029	1.1729	0.1171	0.086*	

### Atomic displacement parameters (Å^2^)

**Table:**

	*U*^11^	*U*^22^	*U*^33^	*U*^12^	*U*^13^	*U*^23^
Cu1	0.0277 (3)	0.0340 (3)	0.0441 (3)	0.00547 (15)	0.0132 (2)	−0.00158 (17)
Cl1	0.0482 (6)	0.0432 (5)	0.0407 (5)	0.0035 (4)	0.0094 (4)	−0.0023 (4)
N2	0.0355 (16)	0.0317 (15)	0.0418 (16)	0.0041 (12)	0.0118 (13)	−0.0026 (12)
N3	0.0398 (17)	0.0480 (19)	0.0432 (17)	0.0156 (14)	0.0152 (14)	0.0003 (14)
N4	0.0385 (16)	0.0366 (16)	0.0392 (16)	0.0031 (12)	0.0107 (13)	0.0023 (12)
O2	0.0440 (16)	0.0712 (19)	0.0731 (19)	0.0207 (14)	0.0327 (14)	0.0047 (15)
C1	0.040 (2)	0.044 (2)	0.049 (2)	0.0096 (16)	0.0142 (17)	0.0009 (16)
N1	0.0286 (16)	0.0516 (19)	0.0505 (18)	−0.0040 (13)	0.0147 (14)	−0.0005 (14)
C11	0.052 (2)	0.0372 (19)	0.032 (2)	0.0057 (16)	0.0044 (17)	−0.0001 (15)
C5	0.052 (2)	0.038 (2)	0.040 (2)	0.0022 (16)	0.0157 (18)	−0.0024 (16)
O1	0.0326 (15)	0.077 (2)	0.092 (2)	−0.0025 (13)	0.0241 (15)	−0.0042 (17)
C2	0.051 (2)	0.066 (3)	0.059 (3)	0.029 (2)	0.019 (2)	0.002 (2)
C9	0.069 (3)	0.058 (3)	0.058 (3)	−0.024 (2)	0.009 (2)	0.009 (2)
C7	0.041 (2)	0.051 (2)	0.047 (2)	−0.0006 (17)	0.0166 (17)	0.0022 (17)
C12	0.052 (2)	0.044 (2)	0.040 (2)	0.0195 (18)	0.0113 (18)	−0.0006 (17)
C6	0.045 (2)	0.045 (2)	0.056 (2)	−0.0115 (17)	0.0177 (18)	−0.0050 (18)
C10	0.071 (3)	0.036 (2)	0.053 (2)	−0.0020 (19)	0.002 (2)	0.0001 (18)
C8	0.052 (3)	0.067 (3)	0.057 (3)	−0.014 (2)	0.016 (2)	0.006 (2)
C4	0.080 (3)	0.031 (2)	0.079 (3)	0.0027 (19)	0.025 (3)	−0.0044 (19)
C3	0.088 (4)	0.044 (3)	0.084 (3)	0.033 (2)	0.029 (3)	−0.004 (2)

### Geometric parameters (Å, °)

**Table:**

Cu1—N3	1.984 (3)	C5—C4	1.371 (5)
Cu1—N1	2.012 (3)	C5—C6	1.451 (5)
Cu1—N2	2.029 (3)	O1—H1B	0.8200
Cu1—N4	2.072 (3)	C2—C3	1.352 (6)
Cu1—Cl1	2.4316 (10)	C2—H2A	0.9300
N2—C1	1.338 (4)	C9—C8	1.354 (6)
N2—C5	1.340 (4)	C9—C10	1.385 (6)
N3—C12	1.256 (5)	C9—H9A	0.9300
N3—O2	1.341 (3)	C7—C8	1.361 (5)
N4—C7	1.335 (4)	C7—H7A	0.9300
N4—C11	1.350 (4)	C12—H12A	0.9300
C1—C2	1.370 (5)	C6—H6A	0.9300
C1—H1A	0.9300	C10—H10A	0.9300
N1—C6	1.258 (4)	C8—H8A	0.9300
N1—O1	1.345 (3)	C4—C3	1.371 (6)
C11—C10	1.375 (5)	C4—H4A	0.9300
C11—C12	1.453 (5)	C3—H3A	0.9300
			
N3—Cu1—N1	91.79 (14)	C4—C5—C6	123.0 (4)
N3—Cu1—N2	168.29 (12)	N1—O1—H1B	109.5
N1—Cu1—N2	79.19 (11)	C3—C2—C1	118.4 (4)
N3—Cu1—N4	79.15 (12)	C3—C2—H2A	120.8
N1—Cu1—N4	151.07 (12)	C1—C2—H2A	120.8
N2—Cu1—N4	105.21 (11)	C8—C9—C10	118.3 (4)
N3—Cu1—Cl1	94.60 (9)	C8—C9—H9A	120.9
N1—Cu1—Cl1	107.40 (9)	C10—C9—H9A	120.9
N2—Cu1—Cl1	95.22 (8)	N4—C7—C8	124.0 (4)
N4—Cu1—Cl1	100.71 (8)	N4—C7—H7A	118.0
C1—N2—C5	118.1 (3)	C8—C7—H7A	118.0
C1—N2—Cu1	128.8 (2)	N3—C12—C11	116.1 (3)
C5—N2—Cu1	112.8 (2)	N3—C12—H12A	121.9
C12—N3—O2	120.3 (3)	C11—C12—H12A	121.9
C12—N3—Cu1	117.1 (2)	N1—C6—C5	115.7 (3)
O2—N3—Cu1	122.1 (2)	N1—C6—H6A	122.2
C7—N4—C11	117.2 (3)	C5—C6—H6A	122.2
C7—N4—Cu1	131.7 (2)	C11—C10—C9	119.9 (4)
C11—N4—Cu1	110.9 (2)	C11—C10—H10A	120.0
N2—C1—C2	122.9 (4)	C9—C10—H10A	120.0
N2—C1—H1A	118.5	C9—C8—C7	119.2 (4)
C2—C1—H1A	118.5	C9—C8—H8A	120.4
C6—N1—O1	118.2 (3)	C7—C8—H8A	120.4
C6—N1—Cu1	116.6 (2)	C5—C4—C3	119.3 (4)
O1—N1—Cu1	125.2 (2)	C5—C4—H4A	120.4
N4—C11—C10	121.4 (4)	C3—C4—H4A	120.4
N4—C11—C12	115.3 (3)	C2—C3—C4	119.8 (4)
C10—C11—C12	123.2 (3)	C2—C3—H3A	120.1
N2—C5—C4	121.4 (4)	C4—C3—H3A	120.1
N2—C5—C6	115.6 (3)		

### Hydrogen-bond geometry (Å, °)

**Table:**

*D*—H···*A*	*D*—H	H···*A*	*D*···*A*	*D*—H···*A*
O1—H1B···O2	0.82	1.70	2.488 (5)	162
